# Exploring the Ability of LARS2 Carboxy-Terminal Domain in Rescuing the MELAS Phenotype

**DOI:** 10.3390/life11070674

**Published:** 2021-07-10

**Authors:** Francesco Capriglia, Francesca Rizzo, Giuseppe Petrosillo, Veronica Morea, Giulia d’Amati, Palmiro Cantatore, Marina Roberti, Paola Loguercio Polosa, Francesco Bruni

**Affiliations:** 1 Department of Biosciences, Biotechnologies and Biopharmaceutics, University of Bari Aldo Moro, 70125 Bari, Italy; francesco.capriglia@uniba.it (F.C.); francesca.rizzo1@uniba.it (F.R.); palmiro.cantatore@uniba.it (P.C.); marina.roberti@uniba.it (M.R.); 2 Institute of Biomembranes, Bioenergetics and Molecular Biotechnologies (IBIOM), National Research Council (CNR), 70125 Bari, Italy; g.petrosillo@ibbe.cnr.it; 3 Institute of Molecular Biology and Pathology (IMBP), National Research Council (CNR), 00185 Rome, Italy; veronica.morea@cnr.it; 4 Department of Radiological, Oncological and Pathological Sciences, Sapienza University of Rome, 00185 Rome, Italy; giulia.damati@uniroma1.it; 5 Consorzio Interuniversitario Biotecnologie (CIB), 34149 Trieste, Italy

**Keywords:** Cterm, MELAS, transmitochondrial cybrids, aminoacyl-tRNA synthetases, LARS2, mitochondrial disease, therapeutic peptides

## Abstract

The m.3243A>G mutation within the mitochondrial mt-tRNALeu^(UUR)^ gene is the most prevalent variant linked to mitochondrial encephalopathy with lactic acidosis and stroke-like episodes (MELAS) syndrome. This pathogenic mutation causes severe impairment of mitochondrial protein synthesis due to alterations of the mutated tRNA, such as reduced aminoacylation and a lack of post-transcriptional modification. In transmitochondrial cybrids, overexpression of human mitochondrial leucyl-tRNA synthetase (LARS2) has proven effective in rescuing the phenotype associated with m.3243A>G substitution. The rescuing activity resides in the carboxy-terminal domain (Cterm) of the enzyme; however, the precise molecular mechanisms underlying this process have not been fully elucidated. To deepen our knowledge on the rescuing mechanisms, we demonstrated the interactions of the Cterm with mutated mt-tRNALeu^(UUR)^ and its precursor in MELAS cybrids. Further, the effect of Cterm expression on mitochondrial functions was evaluated. We found that Cterm ameliorates de novo mitochondrial protein synthesis, whilst it has no effect on mt-tRNALeu^(UUR)^ steady-state levels and aminoacylation. Despite the complete recovery of cell viability and the increase in mitochondrial translation, Cterm-overexpressing cybrids were not able to recover bioenergetic competence. These data suggest that, in our MELAS cell model, the beneficial effect of Cterm may be mediated by factors that are independent of the mitochondrial bioenergetics.

## 1. Introduction

Mitochondrial encephalomyopathies are a group of complex and clinically heterogeneous metabolic disorders caused by a wide spectrum of mutations either in mitochondrial DNA (mtDNA) or in nuclear genes encoding proteins that control mitochondrial functions [1]. Such genetic defects predominantly affect the mitochondrial oxidative phosphorylation system (OXPHOS), which is responsible for most of the ATP supply in cells. OXPHOS is located in the mitochondrial inner membrane and consists of five complexes, denoted as complex I–V. Complex I, III, and IV generate a proton-motive force across the inner membrane, which is used by F1Fo-ATP synthase (complex V) to synthesize ATP. Only 13 subunits of the OXPHOS system are encoded by mtDNA, together with 2 rRNAs and 22 tRNAs necessary for mitochondrial translation machinery. The rest of the OXPHOS subunits, as well as the other proteins that constitute the vast majority of the mitochondrial proteome, are nucleus-encoded and transported into mitochondria [2,3].

Mutations in the mt-tRNA genes are associated with several mitochondrial pathologies. An example is the well-documented mitochondrial encephalopathy with lactic acidosis and stroke-like episodes (MELAS) syndrome. MELAS patients typically develop encephalopathy, stroke-like episodes before the age of 40, and lactic acidosis. Additional clinical features involve other neurological symptoms, exercise intolerance, cardiomyopathy, deafness, and diabetes. About 80% of MELAS syndromes are caused by the m.3243A>G mutation in the mt-tRNALeu^(UUR)^ gene [4,5,6]. The base substitution causes destabilization of the mutated mt-tRNALeu^(UUR)^, thus adversely affecting stability, aminoacylation, and the addition of a taurinomethyl group to the wobble uridine (τm^5^U34) [7,8]. These defects contribute to the overall reduction in mitochondrial protein synthesis observed in MELAS disease [9,10,11].

Despite the increasing knowledge on MELAS pathogenesis, an effective therapy for this and other mitochondrial diseases is still far from being available. As a possible strategy to overcome defects of mt-tRNAs, several molecular approaches have been proposed. One of these consists of the overexpression of human mitochondrial cognate or non-cognate aminoacyl-tRNA synthetases (aaRSs), which proved to rescue the defective viability and energetic competence as well as mitochondrial protein synthesis in transmitochondrial cybrid cell lines, a well-established cellular model of mt-tRNA mutations [12,13]. It was shown in MELAS cybrids that the rescuing activity of human mitochondrial leucyl-tRNA synthetase (LARS2) resided in the non-catalytic carboxy-terminal domain (Cterm, 67 residues long). A more detailed analysis ascribed the rescue capacity of the Cterm fragment to two short β-strand regions, denoted as peptide β30_31 (15 residues in length) and peptide β32_33 (16 residues in length). The correction activity was observed following both overexpression and exogenous administration to cells carrying the m.3243A>G MELAS mutation in the mt-tRNALeu^(UUR)^, as well as the m.8344A>G MERRF mutation in mt-tRNALys. To explain the rescuing effect of the Cterm and its peptides, a chaperonic activity towards mutated tRNAs were proposed on the basis of in vitro evidences, which could result in the stabilization of a wild-type-like conformation of the tRNA [14,15,16,17].

In view of the development of a therapeutic strategy based on the use of Cterm-related small molecules, we decided to deepen our knowledge on the mechanisms that underlie the reported rescuing activity of the Cterm by using the cybrid system. Here, we provide evidence that the overexpressed Cterm domain is able to contact the cognate mutated tRNA in cultured cells, thus corroborating the “chaperonic” hypothesis previously formulated mainly on the basis of in vitro findings. We also evaluated the effect of Cterm overexpression on a range of mitochondrial processes such as mitochondrial translation, steady-state level and aminoacylation efficiency of mt-tRNALeu^(UUR)^, bioenergetics, and mitophagy. In agreement with previous findings [14,15,16,17], a complete recovery of cell viability was observed. Unexpectedly, despite an improvement of protein synthesis, the other analysed mitochondrial processes were not affected by Cterm expression.

## 2. Materials and Methods

### 2.1. Tissue Culture, Transfections and Viability Assay

For RNA immunoprecipitation (RIP) experiments, we used previously established osteosarcoma-derived (143B.TK^−^) cybrid cell lines from MELAS patients and controls [16]. For all the other experiments, we used MELAS cells provided by Prof. A. Filipovska (University of Western Australia). Cybrids were cultured (37 °C, humidified 5% CO_2_) in Dulbecco’s Modified Eagle’s Medium (DMEM, Lonza, Basel, Switzerland) supplemented with 10% (*v*/*v*) foetal bovine serum (FBS, ThermoFisher Scientific, Carlsbad, CA, USA), 1 mM sodium pyruvate, 1× non-essential amino acids, 50 µg/mL uridine, and 1× Antibiotic-Antimycotic (Euroclone, Milan, Italy).

The recombinant expression vector pcDNA6.2 bearing FLAG-tagged Cterm for transient transfections was prepared as follows. The insert was obtained by PCR using the plasmid MTS-Cterm-FLAG [16] as a template and the following primers: For 5′-CACCATGTCCGTCCTGACGCCG-3′ and Rev 5′-CTACTTATCGTCGTCATCCTTGTAATC-3′. The amplicon was cloned into the pcDNA6.2/V5/GW/D-TOPO^®^ vector (Invitrogen, Waltham, MA, USA) according to manufacturer’s instructions. Both Cterm and mock (empty vector) constructs were confirmed by sequencing. Transfections were carried out using the DNA constructs and Lipofectamine™ 3000 Transfection Reagent (ThermoFisher Scientific, Carlsbad, CA, USA) following manufacturer’s recommendations.

For the cell viability assay, cybrids were transfected in 6-well plates and maintained in glucose standard medium. After 24 h of transfection, cells were harvested, seeded at a lower confluency (1.5 × 10^5^ cells/well), and grown for an additional 24 h in either standard medium or galactose medium [glucose-free DMEM (Sigma Aldrich, St. Louis, MO, USA) supplemented with 5 mM galactose, 1% sodium pyruvate, and 10% FBS]. Finally, cells were harvested and counted in triplicate by a TC20TM automated cell counter (Bio-Rad, Hercules, CA, USA) in the presence of trypan blue solution (Sigma Aldrich, St. Louis, MO, USA).

### 2.2. Heteroplasmy Determination by PCR/RFLP Analysis

Total DNA was extracted from the cybrids using the Wizard^®^ Genomic DNA Purification Kit (Promega, Madison, WI, USA) following manufacturer’s recommendations. For RFLP analysis, the mtDNA 3243 locus was amplified using primers flanking the mutation, as follows: For 5′-CCTCGGAGCAGAACCCAACCT-3′ and Rev 5′-CGAAGGGTTGTAGTAGCCCGT-3′. PCR products were digested by Apa I, separated by a 1.5% agarose gel, and stained with GelRed^®^ (Biotium, Fremont, CA, USA). Heteroplasmy levels were determined as a proportion of mutant (digested) to wild type (undigested) mtDNA and measured by ImageQuant TL software (GE Healthcare Life Sciences, Marlborough, MA, USA).

### 2.3. RNA Extraction and RT-qPCR

Total and immunoprecipitated RNA was extracted with TRIsure™ reagent (Meridian Bioscience, Cincinnati, OH, USA) and concentration was measured by NanoDrop™ 1000 (ThermoFisher Scientific, Carlsbad, CA, USA). For RT-qPCR, RNA samples were reverse-transcribed using the High-Capacity cDNA Reverse Transcription Kit (ThermoFisher Scientific, Carlsbad, CA, USA), according to the manufacturer’s instructions. Primers, as reported in Table 1, were designed using the NCBI Primer-BLAST tool; amplification reactions were performed using the SsoAdvanced Universal SYBR^®^ Green Supermix (Bio-Rad, Hercules, CA, USA) and analysed with the Applied Biosystem 7500 Fast Real-Time PCR System (ThermoFisher Scientific, Carlsbad, CA, USA). Relative quantification of the qPCR products was achieved by comparative Ct method; statistical analysis was performed using the unpaired two-tailed Student’s *t*-test.

### 2.4. Whole Cell Extracts Preparation and Mitochondria Isolation

Whole cell extracts were obtained by solubilization in RIPA buffer (25 mM Tris-HCl, pH 7.6; 150 mM NaCl; 1% NP-40; 1% sodium deoxycholate; 1% SDS) with protease inhibitor cocktail (Sigma Aldrich, St. Louis, MO, USA). Extracts were cleared by 5 min centrifugation at 10,000× *g*, snap-frozen, and stored at −80 °C.

The mitochondria were isolated using differential centrifugation. Briefly, confluent cells (6–10 × 10^7^) were washed twice in cold PBS, harvested by scraping, and pelleted at 800× *g* for 7 min at 4 °C. Cells were broken by adding 10 mL of cold mitochondria isolation buffer (MIB: 20 mM HEPES, pH 7.6; 220 mM mannitol; 70 mM sucrose; 1 mM EDTA; 2 mg/mL BSA; 1 mM AEBSF), left on ice for 20 min, and homogenized by 15 strokes using a Thomas homogenizer with a motor-driven Teflon pestle. Homogenate was centrifuged at 800× *g* for 5 min at 4 °C and the supernatant was subsequently spun at 10,000× *g* for 10 min at 4 °C. Crude mitochondrial pellets were washed by suspending in 20 mL of MIB without BSA and centrifuged at 10,000× *g* for 10 min at 4 °C. The mitochondria were suspended in a small volume of MIB without BSA, and protein concentration was determined by the Bradford protein assay. The mitochondria were snap-frozen in liquid nitrogen and stored at −80 °C.

### 2.5. RNA Immunoprecipitation

The MELAS cybrids were stably transfected with either MTS-Cterm-FLAG construct or an empty vector (mock) [16] (90% confluency), grown in 2 × 500 cm^2^ square dishes (Corning, Corning, NY, USA), washed with PBS, crosslinked in 1% formaldehyde-PBS solution for 10 min at room temperature, and then treated with 0.125 M glycine, pH 7.0, for 5 min. The mitochondria were isolated as described above, using a different mitochondria isolation buffer (10 mM Tris-HCl, pH 7.4; 600 mM mannitol; 1 mM EGTA; 0.1% BSA; 1 mM PMSF).

Mitochondrial proteins (600 μg) were immunoprecipitated by anti-FLAG M2 affinity gel (Sigma Aldrich, St. Louis, MO, USA), as reported elsewhere [18]. After crosslink reversion, immunoprecipitated RNA was extracted as described above; isopropanol precipitation was carried out overnight in the presence of 5 μg of glycogen (Roche, Basel, Switzerland) as a carrier. Mitochondrial RNAs in the immunoprecipitate were identified and quantified by RT-qPCR.

### 2.6. SDS-PAGE, BN-PAGE and Immunoblotting

For SDS-PAGE, whole cell proteins (40–100 μg, depending on the proteins being probed) were solubilized in 1× Laemmli buffer and separated by 12% Tris-Glycine-SDS minigels.

Blue-native PAGE (BN-PAGE) analysis was performed using NativePAGE Novex Bis-Tris Gel System (Invitrogen, Waltham, MA, USA) according to the manufacturer’s recommendations. The mitochondrial pellets (40 μg) were solubilized in 50 μL of the NativePAGE Sample Buffer containing dodecylmaltoside (detergent/protein ratio 6:1) and 1 mM AEBSF. After 20 min on ice, samples were centrifuged at 20,000× *g* for 30 min at 4 °C. Supernatants were supplemented with NativePAGE G250 Sample Additive (concentration in the sample was 1/4th to 1/2nd of the detergent concentration) and 15 μg/lane were fractionated through 3–12% NativePAGE Novex Bis-Tris gel at 4 °C.

For western blotting, gels were electro-transferred at 4 °C for 2 h (OXPHOS subunits) or over-night (all the other proteins) onto polyvinylidene difluoride (PVDF) membranes (Millipore, Burlington, MA, USA). Immunoblotting was performed according to standard techniques. Primary antibodies (all from Abcam, Cambridge, UK; diluted 1:1000 except where differently indicated) were as follows: total OXPHOS antibody cocktail (1:250), anti-BNIP3, anti-BNIP3L/NIX, anti-citrate synthase, anti-SDHA, anti-COX I, anti-ATP5A, anti-UQCRC2, and anti-NDUFA9. Detection was performed with the HRP-conjugated secondary antibody (Bio-Rad, Hercules, CA, USA). Chemiluminescent detection was achieved using Amersham ECL™ Prime Western blotting detection reagent (GE Healthcare Life Sciences, Marlborough, MA, USA) or Clarity Western ECL substrate (Bio-Rad, Hercules, CA, USA); signals were revealed by ChemiDoc MP Imaging System (Bio-Rad, Hercules, CA, USA).

### 2.7. [^35^S]-Labelling of Mitochondrial Translation Products

Exponentially growing cells in a 12-well plate were washed twice with methionine-free DMEM and incubated for 1 h at 37 °C with 300 μCi/mL [^35^S]-methionine (Perkin Elmer, Waltham, MA, USA) in 300 μL of methionine-free DMEM supplemented with 10% dialysed FBS, emetine (100 µg/mL), and cycloheximide (100 µg/mL). After the radioactive pulse, cells were washed twice with phosphate-buffered saline (PBS) and finally dissolved in denaturing buffer (3% SDS; 60 mM Tris-HCl, pH 8; 10 mM sucrose, 2 mM EDTA, 5% β-mercaptoethanol). Aliquots (20 µg) of total cell proteins were separated by 15% SDS-polyacrylamide gels and signals were detected by Typhoon FLA 9500 phosphorimager (GE Healthcare Life Sciences, Marlborough, MA, USA). Assessment of protein loading was achieved by Coomassie blue staining.

### 2.8. High Resolution Northern Blot Analysis

High-resolution northerns were carried out as described in [19] with modifications. Total RNA (5 µg) was separated by 15% polyacrylamide-8 M urea gels and electroblotted to Hybond^®^-N+ nylon membranes (GE Healthcare Life Sciences, Marlborough, MA, USA). After transfer, RNA was crosslinked to the membranes at 37 °C for 1 h with freshly prepared EDAC [1-ethyl-3-(3-dimethylaminopropyl) carbodiimide, Sigma Aldrich, St. Louis, MO, USA] reagent. Probes (Table 2) were labelled with non-radioactive digoxigenin-dUTP using the DIG Oligonucleotide Tailing Kit, 2nd Generation (Roche, Basel, Switzerland), following the manufacturer’s recommendations. Pre-hybridization, hybridization, washing, and anti-DIG-AP (Roche, Basel, Switzerland; 1:10,000) incubation were carried out, as detailed elsewhere [20]. Chemiluminescence signals were detected by ChemiDoc MP Imaging System (Bio-Rad, Hercules, CA, USA).

For mt-tRNALeu^(UUR)^ aminoacylation analysis, the same procedures as above were followed, except that total RNA was dissolved in acidic buffer (10 mM sodium acetate, 1 mM EDTA, pH 5.2) and electrophoresed at 4 °C through acid (pH 5.2) 12% polyacrylamide-8 M urea gels to separate charged and uncharged tRNA pools.

### 2.9. Complex IV Activity Determination

Mitochondrial cytochrome c oxidase (complex IV) activity was measured spectrophotometrically, essentially as described in [21] with some modifications. Cells were suspended in hypotonic 20 mM potassium phosphate buffer (pH 7.4) and subjected to three cycles of freezing and thawing. Enzymatic activity was measured at 37 °C in an assay mixture composed of 25 mM phosphate buffer (pH 7.0), 0.75 µM n-dodecyl-β-D-maltoside, and 50 µM reduced cytochrome c. The reaction started following the addition of 50 µg of cell protein in a final volume of 1 mL. The specific activity of the enzyme was expressed as nmol of cytochrome c oxidized/min per mg of cell protein. The cyanide-insensitive rate of cytochrome c oxidation was measured and subtracted.

### 2.10. Mitochondrial Oxygen Consumption Measurement

The mitochondrial oxygen consumption rate (OCR) was calculated by subtracting non-mitochondrial OCR from cellular OCR. This was measured with a Clark-type oxygen electrode (Hansatech Instruments Ltd., King’s Lynn, UK). Oxygen consumption in intact cybrids was measured at 37 °C in 0.5 mL of fresh culture medium lacking glucose, supplemented with 10% foetal bovine serum and 1 mM sodium pyruvate, using a concentration of 2 × 10^6^ cells/mL. Non-mitochondrial oxygen consumption was measured following the addition of 2 µM rotenone and 1.6 µM antimycin A. Results were expressed as nmol O_2_/min per 10^6^ cells.

### 2.11. Lactate Assay

L-lactate production in cell culture medium was measured by spectrophotometrically monitoring NADH formation following its oxidation to pyruvate in the presence of lactate dehydrogenase (LDH). Briefly, non-transfected and transfected cybrids were grown in 6-well plates, and media were collected and centrifuged at 10,000× *g* for 5 min. Aliquots of the supernatant (4–12 μL) were added to a reaction mixture containing 2.5 mM NAD^+^, 320 mM glycine, pH 9.5, 320 mM hydrazine, and 5.8 U of bovine heart LDH (Sigma Aldrich, St. Louis, MO, USA). Reactions were carried out at 25 °C and followed for approximately 15 min.

## 3. Results

### 3.1. Cterm Domain Interacts with the Mutated mt-tRNALeu^(UUR)^ in MELAS Cybrids

**Figure 1:**
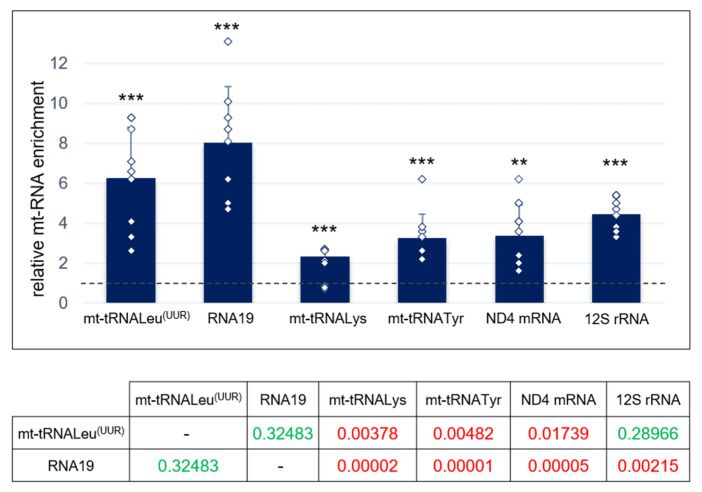
Cterm domain interacts with mutated mt-tRNALeu^(UUR)^ in MELAS cybrids. Relative quantification of mt-RNA species by RT-qPCR was achieved on the Cterm complexes immunoprecipitated with anti-FLAG antibodies from MELAS cybrids stably expressing FLAG-tagged Cterm. Values are referred to immunoprecipitated RNAs obtained from MELAS cybrids stably transfected with empty vector (mock), fixed as 1-value (dashed line). The amount of analysed mt-RNA species in the input was used as endogenous control. Results are presented as the mean ± S.D. Statistical analysis was performed on three independent biological replicates using two-tailed Student’s t test; asterisks (**, *p* < 0.01; ***, *p* < 0.001) indicate the significance of the enrichment of each analysed mt-RNA in Cterm-overexpressing cells with respect to mock. Individual data points are depicted by white diamond-shaped dots. Normal distribution of the six examined mt-RNAs was confirmed by the Shapiro–Wilk test; means were simultaneously compared by one-way ANOVA (α: 0.05). Lower table reports the post-hoc Tukey’s test *p*-values relative to compared pairs of samples (red: statistically significant; green: not statistically significant).

The previously reported rescuing activity of the LARS2 carboxy-terminal domain (Cterm) overexpressed in MELAS mutant cybrids was explained by assuming that Cterm is able to interact with mutated mt-tRNALeu^(UUR)^ [14]. However, such interaction had only been characterised in vitro. Therefore, we decided to investigate the capacity of Cterm to bind mt-tRNALeu^(UUR)^ in cultured cells. In addition, we explored whether this molecule is able to interact with other mitochondrial RNA species, since it is well documented that aaRSs also have the ability to bind non-tRNA substrates [22]. We employed a MELAS cybrid line with a >95% mutation load that stably overexpressed the FLAG-tagged Cterm construct. Firstly, we assessed the Cterm expression by RT-qPCR (Figure S1). Next, we performed RNA immunoprecipitation (RIP) analysis in mitochondrial lysates from MELAS cybrids to identify the RNA species specifically contacted by the Cterm domain. RNA was extracted from complexes immunoprecipitated via anti-FLAG M2 Affinity Gel, and the relative levels of mature mt-tRNALeu^(UUR)^ and its precursor RNA19 (16S rRNA + mt-tRNALeu^(UUR)^ + ND1 mRNA) as well as two mitochondrial tRNAs (mt-tRNALys and mt-tRNATyr), ND4 mRNA and 12S rRNA, were measured by RT-qPCR (Figure 1). In the case of mature mt-RNAs, primers were internal to the mature RNA sequences; for RNA19, primers were designed outside the mt-tRNALeu^(UUR)^, with forward and reverse (see Table 1) placed on the 16S rRNA and ND1, respectively.

We found that the mature and precursor forms of mt-tRNALeu^(UUR)^ exhibited a 6- and 8-fold enrichment, respectively, in immunoprecipitated RNA from Cterm-overexpressing cells with respect to cells transfected with an empty vector (mock) (Figure 1 and Figure S2). An enrichment was also measured for the other examined mitochondrial RNA species, although to a lesser extent. These results clearly demonstrate that: (i) a specific interaction occurs between the Cterm domain and its cognate target tRNALeu^(UUR)^ in MELAS cybrids, in agreement with the direct interaction previously demonstrated to occur in vitro [14]; and (ii) the Cterm also interacts with other mt-tRNAs as well as different species of mt-RNAs.

### 3.2. Cterm Rescues Viability and Mitochondrial Translation in MELAS Cybrids

**Figure 2:**
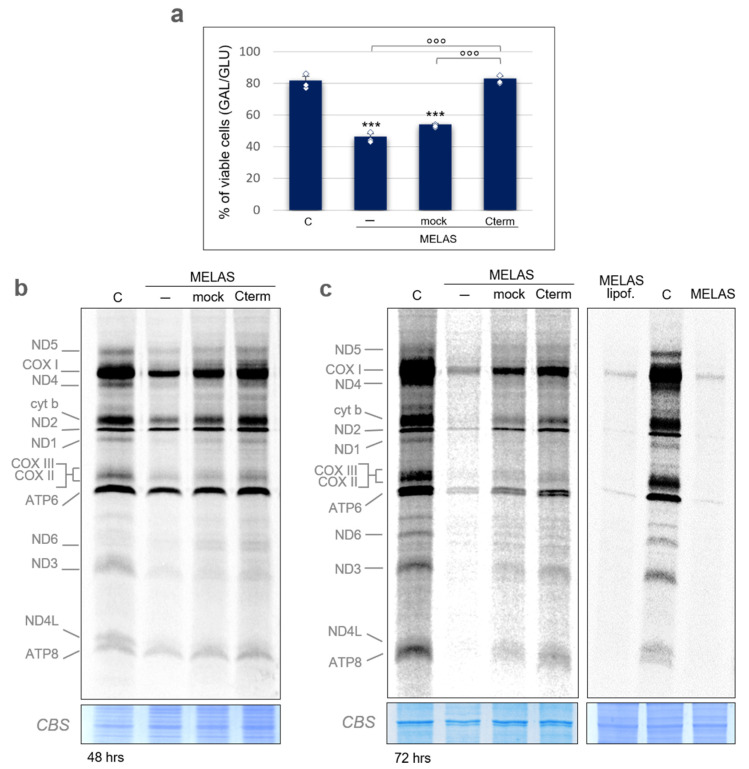
Cterm rescues viability and mitochondrial translation in MELAS cybrids. (**a**) Viability of wild-type control cybrids (C) and MELAS cybrids either non-transfected (-) or transiently transfected with an empty pcDNA6.2 vector (mock) or a Cterm-overexpressing vector (Cterm) evaluated after 24 h-incubation in galactose medium. The number of viable cells in galactose was normalized to the number of viable cells in glucose. Results are presented as the mean ± S.D. Statistical analysis was performed on three independent biological replicates using two-tailed Student’s t test; asterisks (***, *p* < 0.001) indicate the significance of the decreased viability in MELAS cells with respect to the control. Normal distribution of MELAS samples was confirmed by the Shapiro–Wilk test and means were simultaneously compared by one-way ANOVA (α: 0.05) followed by post-hoc Tukey’s test; degree symbols (°°°, *p* < 0.001) refer to the significance of the increase in cybrids overexpressing Cterm with respect to the non-transfected and mock. Individual data points are depicted by white diamond-shaped dots. (**b**) Metabolic [^35^S]-methionine labelling of mitochondrial translation products performed in wild-type cybrids (C) and in MELAS cybrids detailed in (**a**). Pulse-labelling (1 h) was carried out 48 h after transfection; total cell protein (20 µg) were separated by 15% SDS-PAA gels. Mitochondrially encoded polypeptides were assigned, as in [24]. Coomassie blue staining (CBS) of the gel was used as loading control. (**c**) Metabolic [^35^S]-methionine labelling as in (**b**), except that labelling was performed 72 h after transfection (left panel). Labelling of MELAS cells treated with the transfection reagent only (lipof.) was also carried out (right panel).

It is known that, in the vast majority of the cases, the MELAS 3243A>G mutation results in a molecular phenotype characterized by a defective mitochondrial protein synthesis [9,10,11]. Therefore, we decided to assess the impact of Cterm peptide on mitochondrial translation efficiency. Since the cybrid lines used for RIP experiments and in previous work [14,15,16,17] do not show defects in mitochondrial protein synthesis, from here on we employed a MELAS cybrid cell line (kindly provided by Prof. A. Filipovska, University of Western Australia) that displays a depressed mitochondrial translation phenotype (see below). In these cells, mtDNA heteroplasmy analysis indicated a 3243A>G mutation load of ~94% (Figure S3). As a first step, we tested how this system of MELAS cybrids responded to Cterm overexpression in terms of cell viability. The experiment was designed as follows. Cells were routinely propagated on glucose; non-transfected (wild-type or control and MELAS) and transiently transfected MELAS cells (empty vector or mock, and Cterm-FLAG overexpressing vector or Cterm) were transferred in either glucose or galactose medium. The latter forces cells to rely on the mitochondrial respiration for ATP synthesis through pyruvate oxidation because conversion of galactose to pyruvate yields no net ATP. On the contrary, the production of pyruvate via glycolytic metabolism of glucose yields two net ATP molecules [23]. Viable cells were counted after 24 h. As expected, a significant growth decline in galactose was observed for MELAS cells as compared to wild-type cybrids (Figure 2a). Most importantly, MELAS cybrids transfected with the Cterm-expressing vector showed a significant increase in viability with respect to mock and non-transfected cells. The restored viability was comparable to that of wild-type cells. For each transfection experiment, Cterm expression was assessed by RT-qPCR (Figure S4). These results indicate that Cterm is able to rescue the cell viability in line with what was previously reported on different MELAS cybrid cell lines [14,16].

To assess the Cterm effect on de novo synthesis of mtDNA-encoded polypeptides, protein labelling was performed on 48- and 72-h transfected cells in the presence of [^35^S]-methionine and inhibitors of cytosolic translation cycloheximide and emetine. For the 72-h transfection, cells were subjected to additional stress by not replacing the growth medium; this caused medium acidification and accumulation of cellular metabolism by-products.

In all of these experiments, the protein labelling pattern of wild-type cells showed the typical profile of mitochondrial products (Figure 2b,c, Figures S5 and S6). A general consistent decrease in protein synthesis, which was much more pronounced after 72-h transfection, was observed in MELAS cybrids (Figure 2b,c and Figure S7). Interestingly, Cterm transient overexpression was able to significantly rescue most of the protein synthesis defect observed in MELAS cells. Unexpectedly, mock cells exhibited a partial recovery of the newly synthesized polypeptide level. The mock stimulating effect does not seem to depend on the pcDNA6.2 plasmid since two different wild-type plasmids, namely pBAD and pQE60, caused a similar increase (Figure S8). However, transfection with the vector-bearing Cterm construct clearly induced a better recovery of the deficit observed in MELAS cybrids. Finally, to rule out any stimulating effect of the transfection reagent, we performed a protein labelling experiment in the presence of lipofectamine only, showing no effect (Figure 2c, right panel).

### 3.3. Cterm Has No Effect on mt-tRNALeu^(UUR)^ Steady-State Level and Aminoacylation

**Figure 3:**
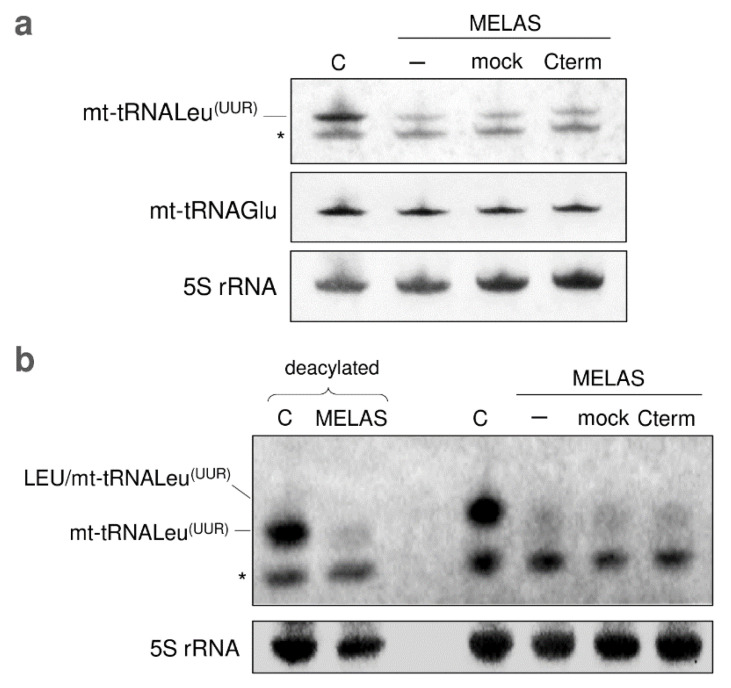
Cterm has no effect on mt-tRNALeu^(UUR)^ steady-state level and aminoacylation. (**a**) Steady-state levels of mt-tRNALeu^(UUR)^ and mt-tRNAGlu in control (C) and MELAS cybrids, either non-transfected (-) or transfected with empty pcDNA6.2 vector (mock) or Cterm-overexpressing vector (Cterm), were determined by high-resolution northern blot analysis with the indicated specific oligonucleotide probes. Each of the three DIG-labelled oligonucleotides detected a band corresponding to the size expected for mt-tRNALeu^(UUR)^ (78 nt), mt-tRNAGlu (72 nt), and 5S rRNA (120 nt, used as loading control), respectively. The asterisk (*) indicates a non-specific band detected by mt-tRNALeu^(UUR)^ probe. (**b**) The aminoacylation levels of mt-tRNALeu^(UUR)^ in wild-type cybrids (C) and MELAS cybrids [same as described in (a)] were estimated by acidic high resolution northern blot analysis. DIG-labelled oligos, as in (a), were probed to mt-tRNALeu^(UUR)^ and 5S rRNA. Positions of aminoacylated and deacylated tRNA (80 °C for 15 min at pH 8) are indicated on the left and are in agreement with Chomyn et al. [25].

Additional distinctive features of cells carrying the MELAS mutation are the decreased steady-state level and aminoacylation efficiency of mt-tRNALeu^(UUR)^, due to 3243A>G substitution that would cause structural destabilization of the mutated tRNA, making it prone to degradation. Thus, the interaction of Cterm with mt-tRNALeu^(UUR)^ might serve to protect the tRNA against misfolding, preventing its degradation. In view of this, Cterm overexpression in MELAS cybrids would result in a recovery of the mt-tRNALeu^(UUR)^ steady-state level. To test this hypothesis, we measured the mt-tRNALeu^(UUR)^ content by high-resolution northern blot analysis. As shown in Figure 3a, the steady-state level of mt-tRNALeu^(UUR)^ remarkably decreased in MELAS cells, with no recovery effect in the cells transfected with Cterm and mock as well as with pBAD or pQE60 plasmids (Figure S9). As a control, we showed that the level of mt-tRNAGlu was unchanged in all analysed conditions.

Since the observed increase in mitochondrial protein synthesis apparently did not seem to be related to the mt-tRNALeu^(UUR)^ steady-state level, we investigated whether Cterm overexpression could somehow improve the aminoacylation efficiency of mutated mt-tRNALeu^(UUR)^ by rendering the bound tRNA more accessible to the aminoacyl-tRNA synthetase. Our results, however, seem to rule out such a possibility because we did not find any increase in the level of mt-tRNALeu^(UUR)^ aminoacylation in Cterm-overexpressing cybrids (Figure 3b).

### 3.4. Cterm Does Not Affect Mitochondrial Bioenergetic Competence, Mitophagy and Mitochondrial Mass

**Figure 4:**
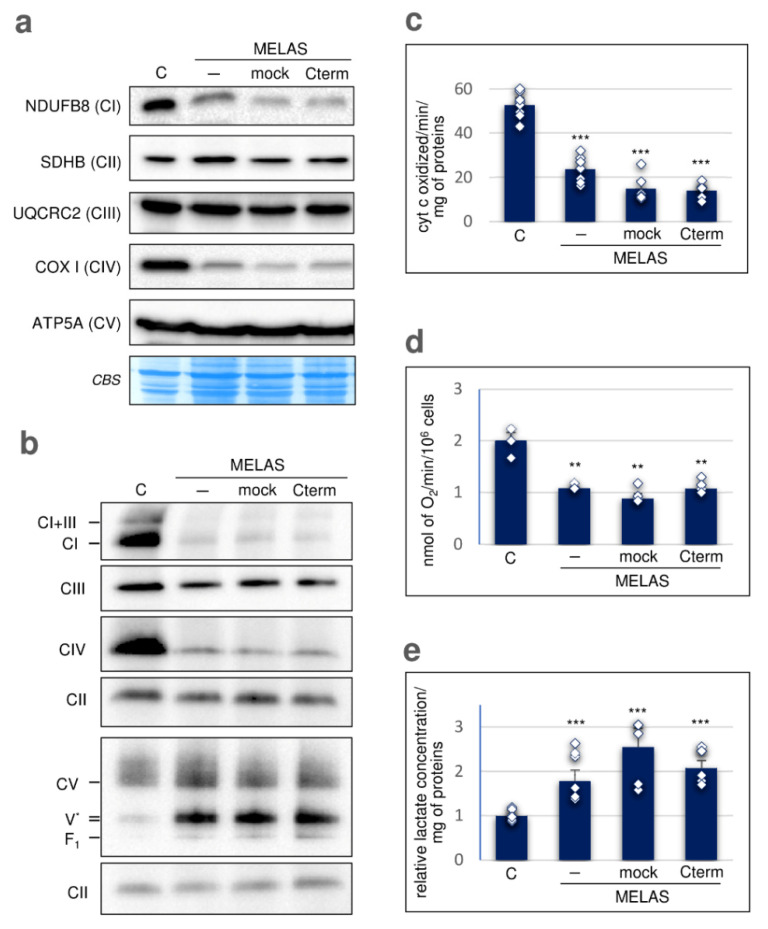
Cterm does not improve mitochondrial bioenergetic competence. (**a**) SDS-PAGE western blot analysis of representative subunits of complex I (NDUFB8), II (SDHB), III (UQCRC2), IV (COX I, mtDNA-encoded) and V (ATP5A or alpha subunit). Immunoblot was carried out using total OXPHOS antibody cocktail (Abcam). Coomassie blue staining (CBS) of the membrane was used as transfer control. (**b**) Blue-native PAGE western blot analysis of the respiratory chain assembled complexes in cybrid cells. Holoenzyme complexes and sub-complexes were visualized by single primary antibodies, as follows: NDUFA9 for complex I, UQCRC2 for complex III, COX I for complex IV, ATP5A for complex V, and SDHA for complex II (used as loading reference). F1-ATPase domain alone (F1) and with several c-subunits (V*) are also indicated. (**c**) Measurement of cytochrome c oxidase (complex IV) activity in whole cybrid cells. Enzymatic activity is expressed as nmol of cytochrome c oxidized/min per mg of proteins. (**d**) Mitochondrial oxygen consumption rate in intact cybrids. Each value is expressed as nmol of O_2_/min per 10^6^ cells. (**e**) Extracellular lactate levels were measured in cell growth media, normalized to total cellular proteins and expressed as fold change relative to control cells (fixed as 1-value). In panels (**c**–**e**), results are presented as the mean ± S.D. and statistical analyses were performed on at least three independent biological replicates using two-tailed Student’s t test; asterisks (**, *p* < 0.01; ***, *p* < 0.001) indicate the significance of variations in MELAS samples with respect to the control. Individual data points are depicted by white diamond-shaped dots. For all the panels, cells were as previously specified.

We then tested whether the observed rescuing effect of the Cterm domain on mitochondrial protein synthesis reflects into any improvement of the oxidative phosphorylation capacity. First, the steady-state level of the five subunits representative of the OXPHOS system was analysed by SDS-PAGE and immunoblotting (Figure 4a and Figure S10, upper panel).

MELAS cells showed a severe decrease in NDUFB8, a nuclear-encoded subunit of complex I and COX I, a mitochondrial-encoded subunit of complex IV, whilst the nuclear-encoded SDHB (complex II), UQCRC2 (complex III) and ATP5A (complex V) were unchanged. Cterm overexpression was unable to rescue the levels of both NDUFB8 and COX I subunits. Then, we measured the levels of the assembled respiratory chain complexes by immunoblotting of crude mitochondrial fractions solubilized with dodecylmaltoside and resolved by BN-PAGE (Figure 4b and Figure S10, lower panel). This analysis showed that complex IV was markedly diminished in MELAS cells, as was complex I alone and in association with complex III (I+III). This is mostly in line with the data reported in literature, showing that combined complexes I-IV deficiency is a general feature of MELAS patients carrying the m.3243A>G mutation [26]. Upon Cterm overexpression, no increase in the content of the affected respiratory complexes was detected, which is in accordance with the immunoblot data relative to the OXPHOS subunits after SDS-PAGE (see Figure 4a). Immunodetection of complex V in wild-type control cells using antibodies against subunit alpha (ATP5A) revealed the presence of complex V holoenzyme along with a very small amount of F1-containing sub-complexes, most likely V* (F1-ATPase with several c-subunits) and F1-ATPase domain alone (Figure 4b). We observed a similar complex V profile in the MELAS mitochondrial fraction, except that a marked increase in the sub-complex V* was found. Furthermore, Cterm overexpression did not cause any change in the complex V profile in MELAS cells.

We assessed COX activity in control and MELAS cells by spectrophotometric assay (Figure 4c), detecting a decrease in MELAS cybrids as expected, given the strong decline in assembled CIV (see Figure 4a). Following overexpression of Cterm, there was no recovery of COX activity in MELAS cells. In line with these data, the mitochondrial oxygen consumption rate of Cterm-transfected cybrids did not increase (Figure 4d); furthermore, lactate production, which was higher in MELAS cells given their prevalent glycolytic metabolism, was not affected by Cterm overexpression (Figure 4e).

**Figure 5:**
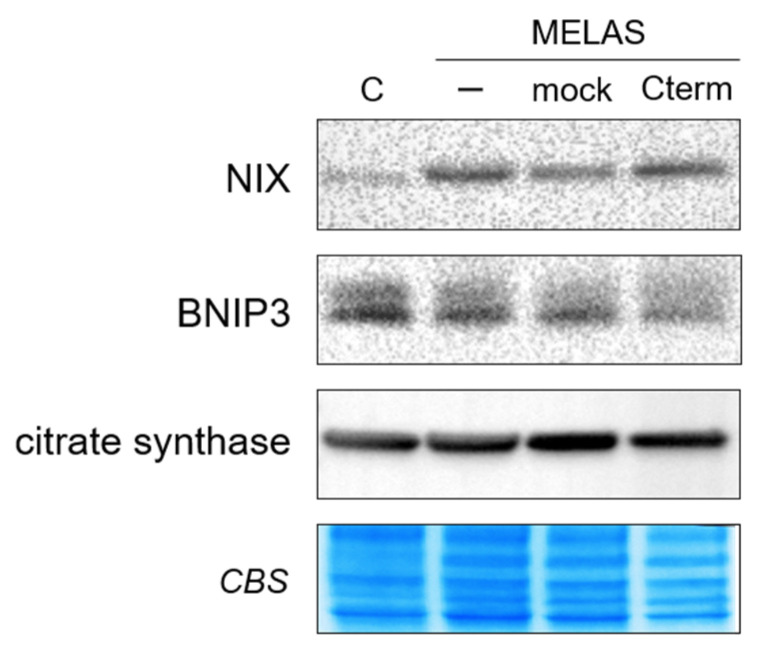
Cterm does not affect mitophagy and mitochondrial mass. Western blot analysis of NIX, BNIP3 and citrate synthase was performed on whole cell lysate. Coomassie blue staining (CBS) of the membrane was used as a loading and transfer control.

To further asses the biochemical effect of Cterm on transfected cybrids, we evaluated mitochondrial degradation in control and MELAS cells by examining the levels of mitophagy receptors BNIP3 and BNIP3-like (BNIP3L)/NIX (Figure 5). The results showed a marked NIX increase in MELAS non-transfected cells with respect to controls, in agreement with previous results, showing that mitophagy activation occurs in MELAS cells to eliminate dysfunctional mitochondria [27,28]. The expression of BNIP3, instead, remained almost unvaried in MELAS cells as compared to controls, suggesting a little impact of this receptor on mitophagy in our MELAS cybrids. However, Cterm overexpression did not substantially alter the expression level of either protein, which remained unchanged with respect to non-transfected MELAS cells. We also examined mitochondrial mass by immunoblot analysis of citrate synthase amount, a mitochondrial matrix enzyme whose level correlates with mitochondrial mass. We found that citrate synthase levels were similar in control and MELAS cells, and did not change in Cterm-overexpressing cells (Figure 5). Collectively, these findings indicate that Cterm overexpression had no impact on mitophagy and mitochondrial content.

## 4. Discussion

Mitochondrial encephalopathy with lactic acidosis and stroke-like episodes (MELAS) syndrome is among the most studied neurodegenerative mitochondriopathies. In 80% of cases, this pathology is associated with the point mutation A3243G in the mt-tRNALeu^(UUR)^ gene [29,30]. The MELAS mutation disrupts the tertiary structure of the mutant tRNA, resulting in various defects spanning from improper RNA processing with accumulation of a large polycistronic precursor transcript to decreased tRNA stability and both aminoacylation and τm^5^U34 wobble posttranscriptional modification deficiency [31,32,33]. All of these defects are causative factors of impaired mitochondrial translation typical of MELAS syndrome.

Numerous studies performed in yeast [34] and human cell lines [13,14,15,16] highlighted the ability of the LARS2 carboxy-terminal domain (Cterm) to interact with several mt-tRNAs, including the cognate mt-tRNALeu^(UUR)^, and to relieve cell growth and respiration defects due to mt-tRNA mutations. Furthermore, a similar rescuing effect by short Cterm-derived peptides was also observed in human MELAS cybrids [17].

The exact molecular mechanism of this rescue activity remains unclear, although a chaperonic effect by Cterm on the mutated tRNA has been proposed, whereby the Cterm would stabilize a functional tRNA conformation and attenuate the detrimental effects of mt-tRNA mutation. In light of this, we decided to undertake a study aimed at elucidating the molecular effect of Cterm on the mitochondrial function in MELAS cybrid-transfected cells.

Here, as a starting point of our investigation, we showed that overexpressed Cterm was capable to interact with the mutated cognate mt-tRNALeu^(UUR)^ in cultured cybrids; this finding is in line with the obtained in vitro results and proposed chaperonic effect of Cterm domain [14,15,16,17]. Then, we assessed whether Cterm was able to relieve mitochondrial molecular defects in a MELAS cell line characterised by depressed mitochondrial translation. In parallel with a significant increase in cell growth, we observed a mild but consistent enhancement of de novo mitochondrial protein synthesis. The latter result is likely mediated by the observed interaction between Cterm and the mutated mt-tRNALeu^(UUR)^. However, our results indicate that the amelioration of both cell viability and de novo protein synthesis was not paralleled by a detectable increase in either steady-state level or aminoacylation efficiency of mt-tRNALeu^(UUR)^. Failure to increase the level of mt-tRNALeu^(UUR)^ is actually not surprising because previous reports suggested that the chaperonic activity of Cterm would improve the function of mutated tRNALeu^(UUR)^ by stabilizing its shape rather than preventing the degradation [16]. Mitochondrial tRNAs are known to have great structural instability compared to the bacterial counterparts [35], which explains why stabilizing proteins in general can improve mt-tRNA aminoacylation, especially in the presence of destabilizing mutations (as discussed in [13]). In this framework, it is puzzling that we could not detect a clear enhancement of tRNALeu^(UUR)^ aminoacylation by the stabilizing molecule Cterm. One possible explanation is that, since mutated mt-tRNALeu^(UUR)^ levels in MELAS cells are very low (as we show here) and only a fraction of the tRNA would reasonably undergo aminoacylation by the intervention of Cterm, the signal of aminoacylation enhancement would be below the detection limit of the technique we used in this experiment. The improvement of mitochondrial translation driven by the Cterm domain could also be ascribed to the capacity of the peptide to restore wobble taurine modification on the mutated tRNA or to improve interactions with the ribosome or with other proteins required to exert its function.

We showed here that Cterm is able to bind not only the mature mt-tRNALeu^(UUR)^ but also its precursor, named RNA19. This is a polycistronic transcript including 16S rRNA, mt-tRNALeu^(UUR)^, and ND1 mRNA, which accumulates because of processing defects caused by the mutated tRNA. Increased levels of RNA19 have been described in MELAS cells carrying the A3243G mutation [9,36] and also in cybrids bearing the mt-tRNALeu^(UUR)^ A3302G mutation [37]. Attardi and colleagues [25] provided evidence for defective polysome formation in MELAS cells, which could lead to mRNAs degradation resulting in a decreased translation rate. A possible cause of functionally deficient polysomes could be that RNA19 is incorporated into ribosomes rendering them rate-limiting for translation [31]. In light of this, the observed increase in mitochondrial protein synthesis in Cterm-overexpressing cells might be due to the ability of the Cterm to sequester RNA19 and/or promote its correct processing, minimizing the accumulation of such an abnormal RNA processing transcript. This hypothesis is also in agreement with our RIP data, which show a preferential enrichment of RNA19, notwithstanding the higher physiological amount of mature mt-tRNALeu^(UUR)^ with respect to its precursor.

A puzzling finding of our study is that the observed rescuing did not correspond to an improvement in the analysed bioenergetic aspects. Steady-state levels of representative OXPHOS subunits of complex I and IV remained low in the MELAS cybrids overexpressing Cterm, as did the level of assembled complex I and IV. Accordingly, complex IV activity and mitochondrial oxygen consumption rate did not recover in the presence of the Cterm domain. Moreover, Cterm expression did not influence the higher glycolytic flux peculiar of MELAS cells, as shown by lactate measurement. Interestingly, our MELAS cell line displayed a strong accumulation of ATP synthase F1-c subcomplexes, named V*. It was shown in cultured cells [38] that intermediate subcomplexes V* increase when mitochondrial translation is inhibited by doxycycline, indicating that, in order for the subcomplex to evolve to the fully assembled holoenzyme, the mitochondrially encoded subunits ATP6 and ATP8 are needed. The strong increment of subcomplex V* in MELAS cells is in accordance with the observed decreased level of subunits ATP6 and ATP8 (see Figure 2). Moreover, Cterm did not influence the complex V profile either on a quantitative or qualitative basis. The fact that overexpressed Cterm did not improve these bioenergetic defects could be explained by assuming that, although increased, the observed translation efficiency is still below a threshold for respiratory chain improvement. Even a longer timescale of Cterm expression was not able to produce any increment in the OXPHOS subunit level (data not shown). In light of this, the slight increase in mitochondrial protein synthesis observed in mock cells, which is anyhow lower than that caused by Cterm, has a negligible functional significance, especially considering that no recovery of cell viability occurs upon mock transfection. Finally, Cterm peptide had no impact on mitophagy and mitochondrial mass.

It is important to point out that, unlike what has been here described, some of us reported an increase in oxygen consumption using another MELAS cell line transfected with Cterm [14,16]. This discrepancy suggests that distinct MELAS cybrid lines may have peculiar molecular pathways and compensatory mechanisms, in parallel with the various phenotypes observed in MELAS patients with similar levels of heteroplasmy.

Overall, the reported data tend to exclude that Cterm capacity to restore cell viability to the wild type level derives from a direct involvement of the mitochondrial functions analysed herein, suggesting that other mitochondrial pathways, as well as those extra-mitochondrial, that are possibly triggered by the mitochondrial status are involved. Mitochondrial dysfunction can influence nuclear gene expression in mammalian systems by means of retrograde signalling mediated by ROS, Ca^2+^, ADP/ATP, NAD/NADH, and non-coding RNAs [39,40]. A study of transcriptional reprogramming conducted in the MELAS cybrids highlighted various metabolic pathways that are differentially triggered by variations in mtDNA 3243 mutation heteroplasmy [41]. These pathways, which represent both adaptive and maladaptive cellular responses, refer to a series of genes related to glycolysis for energy production, antioxidant and redox regulatory systems, autophagy/mitophagy, apoptosis, chromatin remodelling, and compartment-specific unfolded protein stress response. In this context, nuclear mRNAs or non-coding RNAs of either nuclear or mitochondrial origin, such as processed mt-tRNA fragments exported to the cytoplasm or circular RNAs [40,42,43,44], may represent possible targets of cytosolic RNA-binding proteins for regulation of nuclear gene expression. In addition to mitochondrial retrograde signalling to the cytoplasm, the full recovery of cell viability might be the result of Cterm capacity to bind cytosolic RNA targets. This hypothesis is supported by the observation that: (i) a certain amount of overexpressed Cterm is not imported into mitochondria and remains in the cytosol [13]; and (ii) Cterm interacts also with non-tRNA targets (see Figure 1). This circumstance is in accordance with the hypothesis that the Cterm mode of RNA recognition relies more on structural rather than on specific base–base interactions, as observed in the available 3D structures of whole LeuRS-tRNALeu complexes from bacterial species [45]. This feature is reminiscent of the promiscuous RNA binding mode typical of aaRSs [22,46].

In conclusion, our results on the rescuing ability of Cterm in MELAS cybrids open up to pathways that, in our cellular model, seem to be independent of the mitochondrial bioenergetics. Importantly, the demonstration that Cterm has rescuing activity in MELAS cybrids, in spite of differences in the apparent mechanism of action, strongly supports the therapeutic potential of the Cterm and of molecules derived thereof [17], opening up new perspectives for the treatment of mitochondrial diseases. Further work on additional cellular models, preferably tissue-specific, will be required to fully unveil the molecular basis of Cterm rescuing activity.

## Figures and Tables

**Table 1:** Primer sequences used for RT-qPCR analyses.

Amplicon	Primer Sequence
mt-tRNALeu^(UUR)^	For: GTTAAGATGGCAGAGCCC
	Rev: GAAGAGGAATTGAACCTCTGAC
RNA19	For: TATACCCACACCCACCCAAG
	Rev: GCGATTAGAATGGGTACAAT
mt-tRNALys	For: ATAGGGCCCGTATTTACCCTA
	Rev: ATACGGTAGTATTTAGTTGG
mt-tRNATyr	For: TGGTAAAAAGAGGCCTAACCC
	Rev: ATGGCTGAGTGAAGCATTGG
ND4 mRNA	For: CCATTCTCCTCCTATCCCTCAAC
	Rev: CACAATCTGATGTTTTGGTTAAAC
12S rRNA	For: ACACTACGAGCCACAGCT
	Rev: GCTACACCTTGACCTAACGTC
Cterm	For: AAATTCCTGTGCCCCAACAA
	Rev: CTACTTATCGTCGTCATCCT
18S rRNA	For: GTAACCCGTTGAACCCCATT
	Rev: CCATCCAATCGGTAGTAGCG

**Table 2:** Oligonucleotides used for DIG-northern analyses.

Target	Probe Sequence
mt-tRNALeu^(UUR)^	TATGCGATTACCGGGCTCTGCCATCTTAAC
mt-tRNAGlu	TATTCTCGCACGGACTACAACCACGAC
5S rRNA	GGGTGGTATGGCCGTAGAC

## Data Availability

Not applicable.

## Supplementary Materials

The following are available online at https://www.mdpi.com/article/10.3390/life11070674/s1, Figure S1: Stable expression of Cterm-FLAG transcript, Figure S2: Raw data from RIP experiments, Figure S3: RFLP analysis and quantification of m.3243A>G heteroplasmy in MELAS cybrids, Figure S4: Transient expression of Cterm-FLAG transcript, Figure S5: Gel scans and densitometry data of metabolic [^35^S]-methionine labelling replicates after 48-h transfection, Figure S6: Gel scans and densitometry data of metabolic [^35^S]-methionine labelling replicates after 72-h transfection; Figure S7: Relative quantification of [^35^S]-labelling signals, Figure S8: Effect of pBAD and pQE60 transfection on metabolic [^35^S]-methionine labelling of mitochondrial translation products, Figure S9: Effect of pBAD and pQE60 transfection on mt-tRNALeu^(UUR)^ and mt-tRNAGlu steady-state levels, Figure S10: Relative quantification of SDS-PAGE and BN-PAGE signals.
