# Supplementary material for: Exploring the Ability of LARS2 Carboxy-Terminal Domain in Rescuing the MELAS Phenotype
Source: Life (Basel). 2021 Jul 10;11(7):674. doi: 10.3390/life11070674 (PMC8303833; doi:10.3390/life11070674)
Supplement: Supplementary file 1 [file life-11-00674-s001.zip › life-1289687-supplementary.pdf]

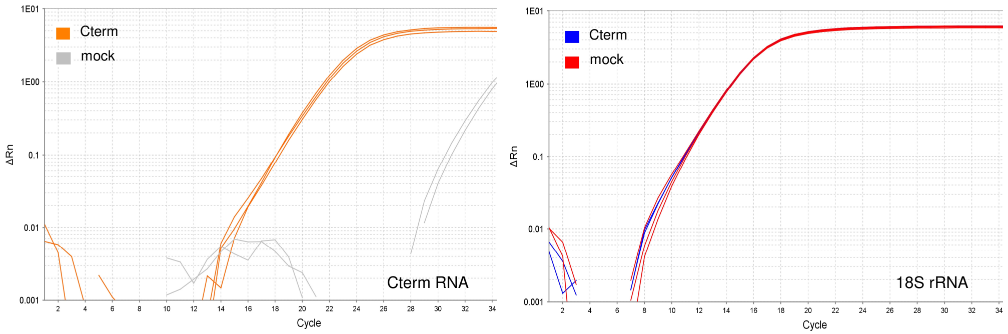

Figure S1. Expression of Cterm-FLAG transcript by RT-qPCR in MELAS cybrids stably transfected with either empty pcDNA3.2 vector (mock) or MTS-Cterm-FLAG vector (Cterm). Nuclear encoded 18S rRNA was used as endogenous control.

	Ratio 1	Ratio 2	Ratio 3	Ratio 4	Ratio 5	Ratio 6	Ratio 7	Ratio 8	Ratio 9
mat.tRNA $_{LEU}$	9.2	9.2	7	4	3.2	2.5	8.6	6.5	6.1
RNA19	13	10	9.2	4.9	4.6	8	8.6	6.1	
tRNA $_{LYS}$	2.6	2.6	2	2.5	1.9				
tRNA $_{TYR}$	3.2	3.5	3.7	2.5	2.5	2.1	3.2	6.1	2.5
ND4	3.5	4	4	1.5	1.9	2.3	6.1	2.3	4.9
12S	5.3	5.3	4.6	3.2	3.7	3.5	4.3	5.3	4.9

	mean	sd	sem	n	p-value
mat.tRNA $_{LEU}$	6.25556	2.54956	0.85	9	*0.0003*
RNA19	8.05	2.81729	1.00	8	*0.0002*
tRNA $_{LYS}$	2.32	0.34205	0.15	5	*0.0001*
tRNA $_{TYR}$	3.25556	1.19385	0.40	9	*0.0005*
ND4	3.38889	1.5227	0.51	9	*0.0015*
12S	4.45556	0.82479	0.27	9	*< 0.0001*

Figure S2. Raw data from RIP experiments in stable MELAS cells. For each mitochondrial RNA, the relative enrichment was measured by RT-qPCR and calculated as ratio between Cterm-overexpressing cybrids and mock cells.

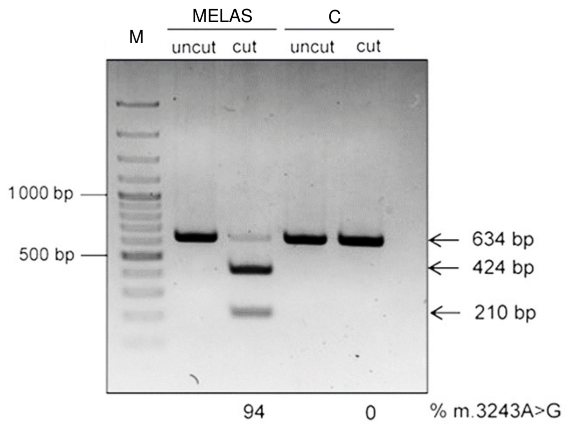

Figure S3. RFLP analysis and quantification of m.3243A>G heteroplasmy in MELAS and wild-type (C) cybrid cells. Digestion was performed with Apa I restriction enzyme and products were separated on 1.5% agarose gel. Mutation load percentage (bottom) was determined as proportion of mutant (424 bp and 210 bp products) to wild type (634 bp product) mtDNA. Band intensities were quantified by ImageQuant TL software (GE Healthcare Life Sciences).

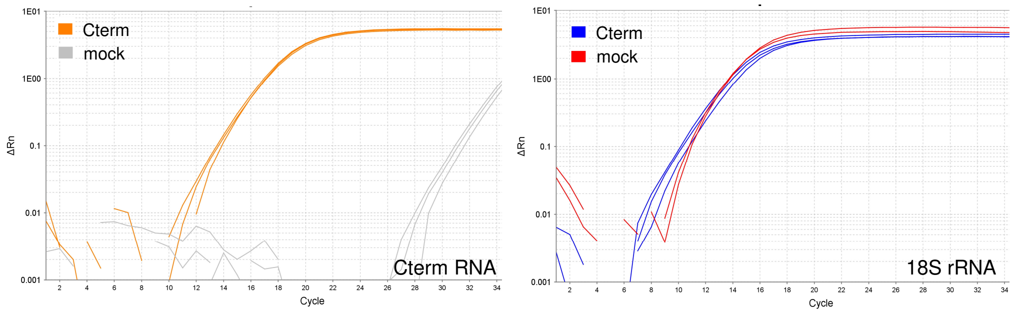

Figure S4. Expression of Cterm-FLAG transcript by RT-qPCR in MELAS cybrids transiently transfected with empty pcDNA6.2 vector (mock) or Cterm-overexpressing vector (Cterm). Nuclear encoded 18S rRNA was used as endogenous control. Shown amplification plots are representative of all the transfection experiments.

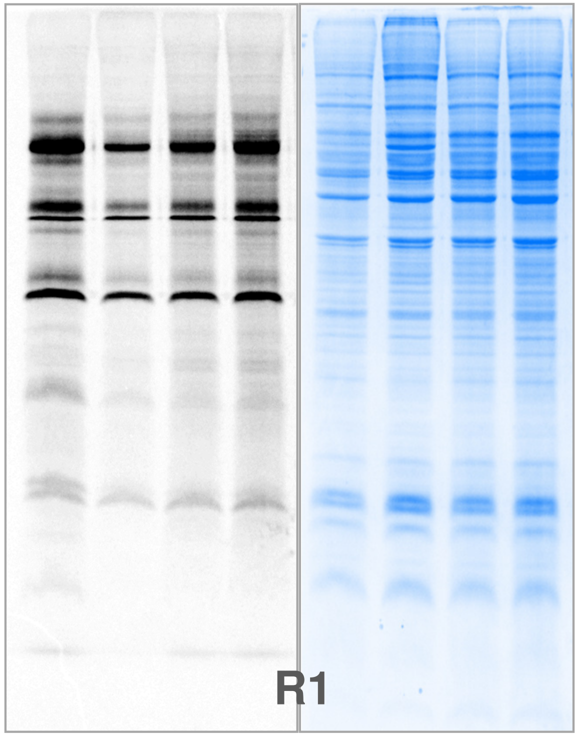

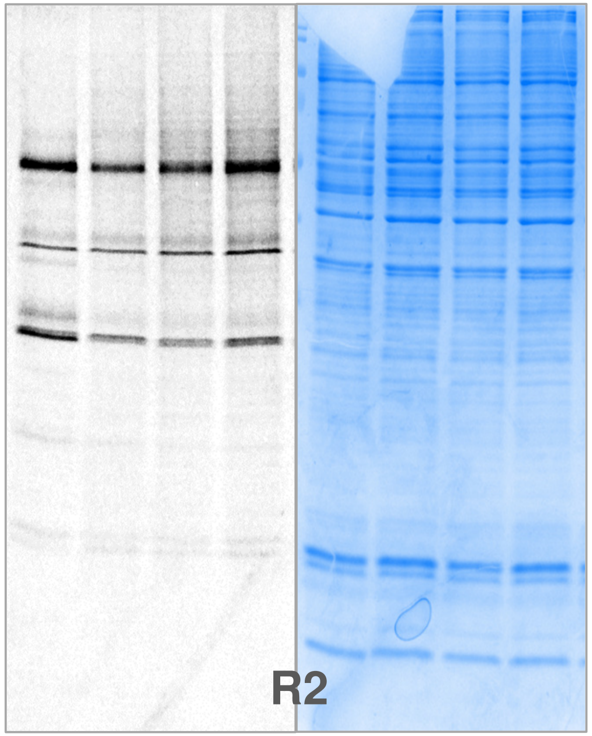

	R1 gel	
1	C	100
2	MELAS	31
3	mock	49
4	Cterm	64

	R2 gel	
1	C	100
2	MELAS	50
3	mock	60
4	Cterm	78

	R3 gel	
1	C	100
2	MELAS	57
3	mock	78
4	Cterm	91

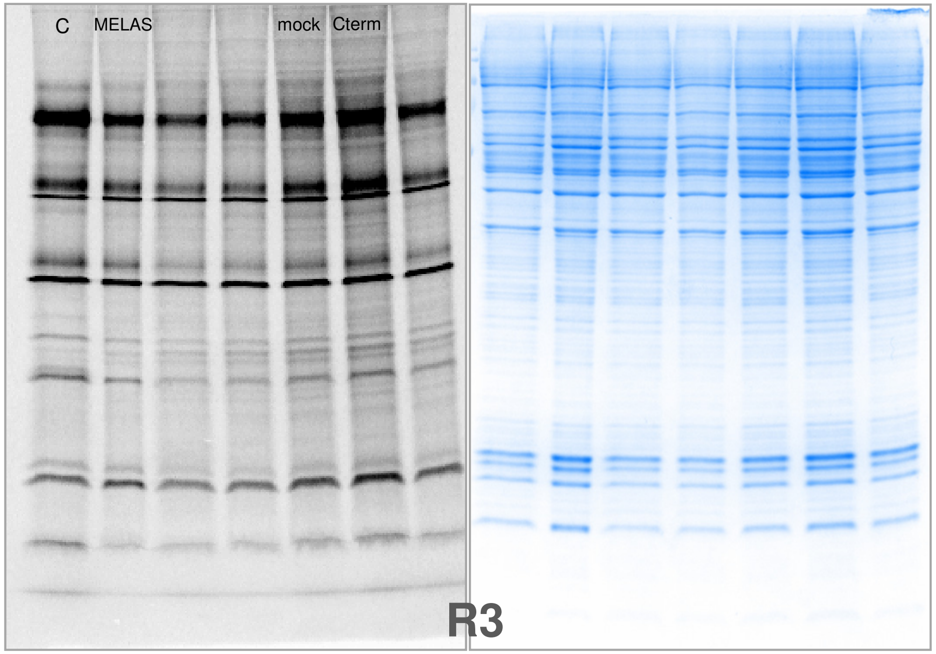

	MEAN	
1	C	**100**
2	MELAS	**46**
3	mock	**62**
4	Cterm	**77.7**

Figure S5. Original scans of metabolic [^{35}S]-methionine labelling replicates (R1-R3) performed in wild-type (C) and MELAS cybrids. Pulse-labelling (1 hour) was carried out 48 hours after transfection; total cell protein (20 µg) were separated by 15% SDS-PAA gels. Coomassie blue staining (CBS) of the gel was used as loading control. For each replicate, quantifications of entire lanes relative to control and MELAS samples, normalized on CBS, are reported on the right.

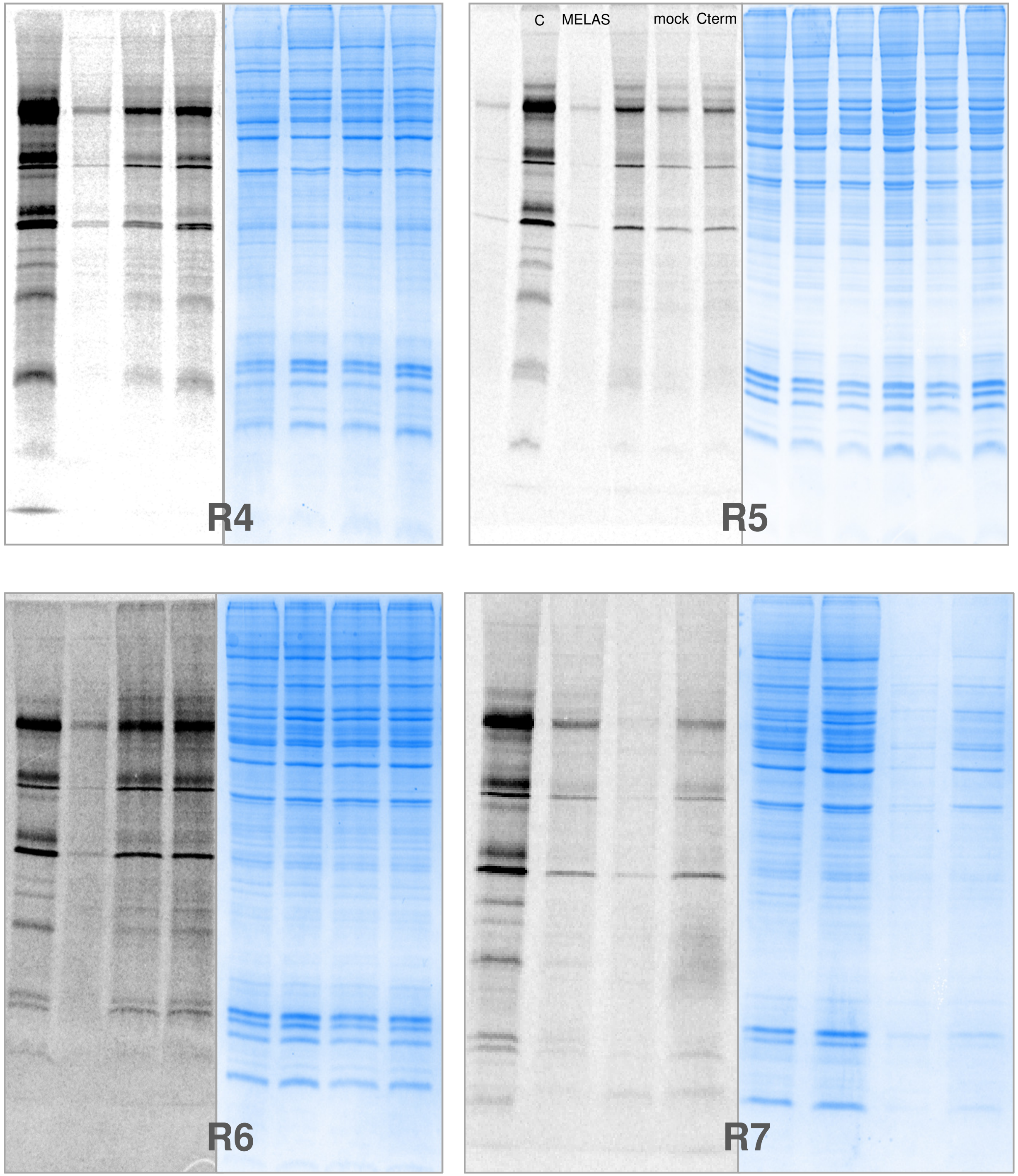

	R4 gel	
1	C	100
2	MELAS	14
3	mock	40
4	Cterm	60

	R5 gel	
1	C	100
2	MELAS	16
3	mock	35
4	Cterm	46

	R6 gel	
1	C	100
2	MELAS	16
3	mock	50
4	Cterm	71

	R7 gel	
1	C	100
2	MELAS	15
3	mock	21
4	Cterm	49

	MEAN	
1	C	**100**
2	MELAS	**15.2**
3	mock	**36.5**
4	Cterm	**56.5**

Figure S6. Original scans of metabolic [^{35}S]-methionine labelling replicates (R4-R7) performed in wild-type (C) and MELAS cybrids. Pulse-labelling (1 hour) was carried out 72 hours after transfection; total cell protein (20 μg) were separated by 15% SDS-PAA gels. Coomassie blue staining (CBS) of the gel was used as loading control. For each replicate, quantifications of entire lanes relative to control and MELAS samples, normalized on CBS, are reported on the right.

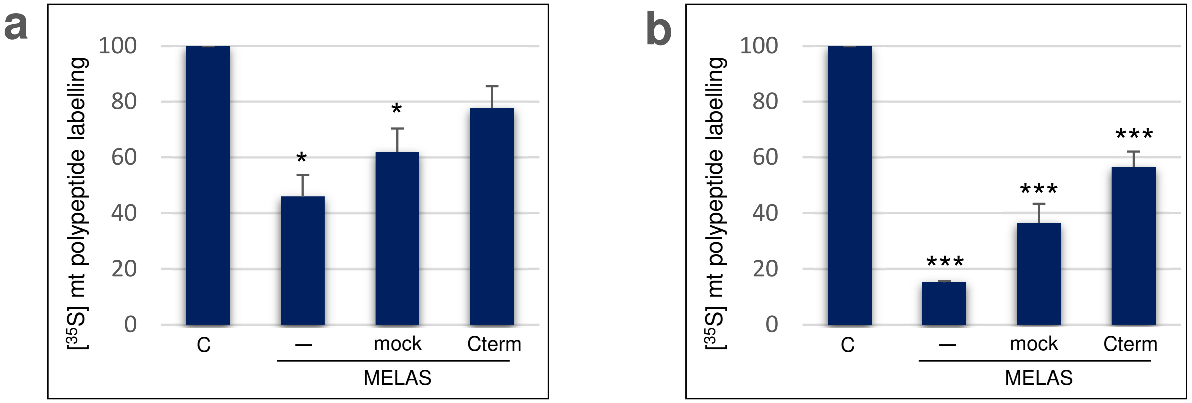

Figure S7. Relative quantification of [^{35}S]-labelling signals after 48-hours transfection (panel **a**) and after 72-hours transfection (panel **b**) was achieved by a densitometric analysis of entire lanes using ImageQuant TL software (GE Healthcare Life Sciences). Data shown in **a)** and **b)** are representative of three (Figure S5) and four (Figure S6**)** independent experiments, respectively (one sample t test: *, $p<0.05$; ***, $p<0.001$).

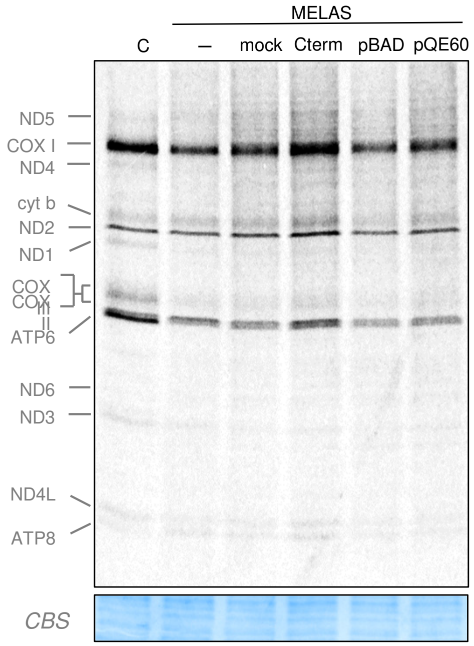

Figure S8. Metabolic [^{35}S]-methionine labelling of mitochondrial translation products performed in control cybrids (C) and in MELAS cybrids either untransfected (-) or transfected with empty pcDNA6.2 vector (mock), Cterm-overexpressing vector (Cterm), pBAD or pQE60 wild-type plasmids. Pulse-labelling (1 hour) was carried out 48 hours after transfection; total cell protein (20 µg) were separated by 15% SDS-PAA gels. Mitochondrially-encoded polypeptides were assigned as in Chomyn et al., 1991 [24]. Coomassie blue staining (CBS) of the gel was used as loading control.

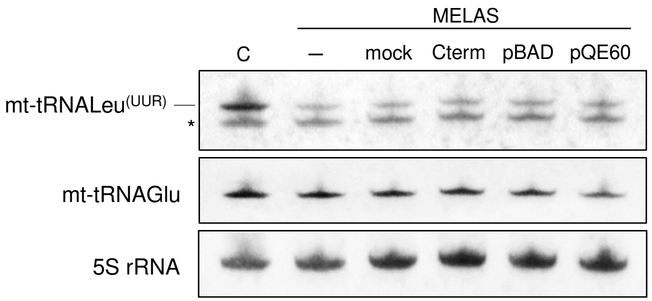

Figure S9. Effect of pBAD and pQE60 transfection on steady-state levels of mt-tRNALeu(UUR) and mt-tRNAGlu in transfected MELAS cells. 5S rRNA was used as loading control. A non-specific band detected by mt-tRNALeu(UUR) probe is indicated with an asterisk. Uncropped image of the gel shown in Figure 3a.

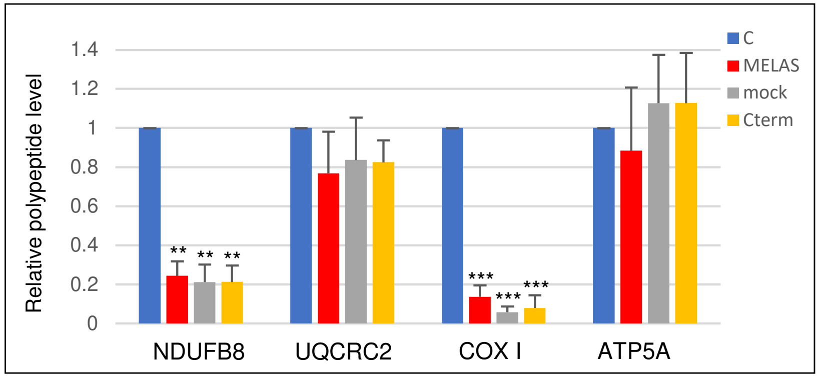

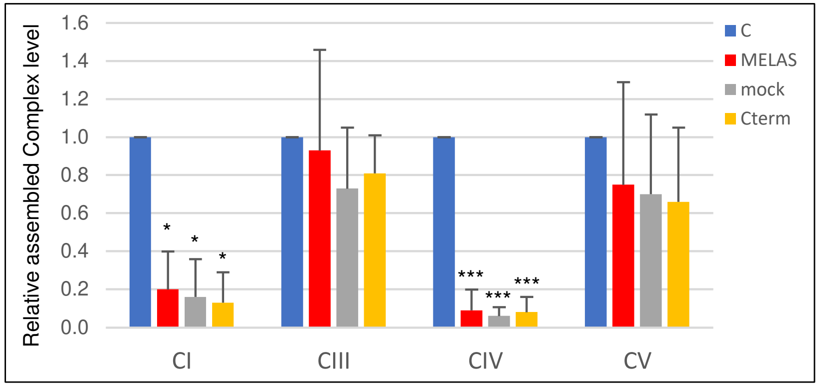

Figure S10. Relative quantification of SDS-PAGE and BN-PAGE signals shown in Figure 4a (upper panel) and in Figure 4b (lower panel), respectively, was achieved by densitometric analysis using ImageQuant TL software (GE Healthcare Life Sciences). Data are representative of three independent experiments (two-tailed Student's t test: *, $p<0.05$; **, $p<0.01$; ***, $p<0.001$).
